# From one tree to the canopy: an evolutionary design trajectory for tethered robotics in forest environments

**DOI:** 10.3389/frobt.2026.1859773

**Published:** 2026-09-16

**Authors:** Luca Romanello, Bahadir Basaran Kocer, Mirko Kovac

**Affiliations:** 1 Laboratory of Sustainability Robotics, EPFL - Ecole Polytechnique Federale de Lausanne, Lausanne, Switzerland; 2 Laboratory of Sustainability Robotics, Empa - Swiss Federal Laboratories for Materials Science and Technology, Duebendorf, Switzerland; 3 School of Civil, Aerospace and Design Engineering, University of Bristol, Bristol, United Kingdom; 4 Aerial Robotics Lab, Department of Aeronautics, Imperial College London, London, United Kingdom

**Keywords:** arboreal locomotion, canopy robotics, environmental robotics, forest sensing, structure-native robotics, sustainable robotics, tethered aerial robots

## Abstract

Forest robotics has largely evolved along a ground-first and flight-dominant trajectory, treating the canopy as a region to observe or briefly access rather than a habitat to inhabit. In this Perspective, we propose an alternative canopy-first framework in which robots operate through anchoring, suspension, and tension-mediated interaction with the environment. We trace the progression from ground and aerial systems to perching mechanisms, identifying their shared limitation as transient canopy interaction. Tether-based robots instead enable sustained presence by transforming branches into structural supports. Within this context, TreeSpider is presented as an early canopy-innate morphology. We argue that such systems point toward a broader class of structure-innate robotics, where behavior emerges through physical coupling with the environment rather than continuous free-space mobility.

## 1 Introduction

### 1.1 Evolution in the treetops

*What if vast areas of tropical rainforest had always been uninhabitable at ground level, whether due to seasonal flooding, toxic decay, or environmental conditions too hostile for sustained life?* In such a scenario, evolution would push organisms to adapt entirely to the canopy, completing their life cycles, feeding, mating, reproducing, and dying, without ever touching the forest floor.

Intense competition for canopy resources would favor organisms capable of persistent attachment, controlled movement through three-dimensional branch networks, and stable positioning under wind and dynamic disturbances. Trees, in turn, might co-evolve stronger branches, increased interconnectivity increased interconnectivity potentially advantageous through shared mechanical load distribution under wind stress Quine and Gardiner (2007) and facilitated seed and nutrient exchange among canopy-dwelling organisms, opening avenues for tree-robot symbiosis, and compliant structures that tolerate frequent interaction. Over generations, this would give rise to lifeforms adapted not for walking or swimming, but for perching, gripping, gliding, and anchoring within a spatially complex arboreal environment. Spiders, the original biological inspiration for tethered robotics Hauf et al. (2023), demonstrate precisely this trajectory: silk begins as a passive anchor and develops into a multifunctional organ for locomotion, prey capture, and environmental interaction, the same arc envisioned for robotic tethers.

In this evolutionary path, we define the tether as an elongated load-bearing appendage independent of the limbs, capable of remote attachment, tensioning, and release, distinct from limb- or tail-based grasping as in spider monkeys. Such an organ could no longer be a secondary tool but a primary one: actuated, adaptive, and multifunctional, inspired not by the liana’s structural role alone but by the broader biological principle of living, growing attachment systems that respond to their environment over time Del Dottore et al. (2024). Retractability, while absent from passive plant growth, is a design aspiration: a step toward tethers that behave less like ropes and more like active appendages. It would enable gap-bridging, stabilization, and locomotion through tension and controlled release. In this scenario, persistence over speed, anchoring over avoidance, and structural interaction over free-space mobility represent one viable strategy among several that could coexist, much as slow persistent climbers and fast gliding escapers share extant canopy ecosystems. This speculative scenario establishes a fundamentally different set of constraints from those that shaped terrestrial or aerial organisms and, by extension, from those that have historically guided robotic system design.

Unlike biological organisms, which carry the irreversible baggage of their evolutionary ancestry, engineered systems can freely discard inherited assumptions and recombine innovations across unrelated lineages. We use the evolutionary framing here to motivate precisely this freedom: canopy robots need not inherit the constraints of ground or aerial design in the way no biological organism ever could.

### 1.2 From canopy ecology to robotic morphology

In a canopy ecosystem, survival favors organisms that can remain in the canopy without continuous energy expenditure, tolerate frequent physical contact, and exploit the redundancy of branches as structural supports. Locomotion is intermittent rather than continuous, emerging from sequences of anchoring, tensioning, release, and reattachment.

If robotic systems were designed under analogous ecological pressures, they would emphasize morphological intelligence over centralized control, persistent anchoring over sustained thrust, and contact-rich locomotion over collision avoidance. However, most robotic systems developed for forest environments have instead inherited assumptions from ground robotics and aviation, reflecting a ground-first and flight-dominant evolutionary trajectory.

To understand why tethered aerial robots represent a departure from this lineage, it is necessary to examine how forest robotics has evolved to date.

## 2 Evolutionary pathways in forest robotics

### 2.1 Forest robotics under a ground-first evolutionary path

The development of forest robotics largely reflects a ground-first evolutionary paradigm, in which systems were designed either to observe forests from above or to incrementally extend terrestrial locomotion upward. The term ground-first refers not to the physical location of these platforms but to their inherited design assumptions: sensors, navigation logics, and operational frameworks developed for terrestrial or open-air environments, projected upward into the canopy rather than conceived for it. While these approaches enabled important advances in environmental sensing, they rarely treated the canopy as a primary habitat to be lived.

#### 2.1.1 Over-canopy observation: remote sensing organisms

Early robotic observation of forests relied predominantly on over-canopy sensing. Satellite imagery and aerial platforms equipped with optical, multispectral, and LiDAR sensors enabled large-scale mapping of canopy height, biomass, and forest dynamics Tian et al. (2020); Hyyppä et al. (2020); Asner and Mascaro (2013). These tools have proven invaluable for monitoring vegetation health, biodiversity indicators, and structural changes over time Mwinuka et al. (2022); Assmann et al. (2018); Raeva et al. (2018).

However, dense foliage severely limits penetration into understory and interior canopy layers, rendering many ecologically relevant processes invisible from above. Consequently, high-resolution in-canopy data collection has remained dependent on manual access methods such as the Single Rope Technique (SRT) Lowman et al. (2012). Mechanized variants, such as cable-riding robots on pre-installed tensioned lines, improve operator safety but still depend on prior human-mediated installation, shifting rather than resolving the access problem. From an evolutionary perspective, these systems resemble remote observers rather than inhabitants of the canopy ecosystem.

#### 2.1.2 Ground and climbing robots: early arboreal analogues

Ground-based robotic systems represented the earliest mechanized attempts to physically access forest environments. Quadrupedal robots demonstrated robust locomotion on uneven forest floors and debris-rich terrain Valsecchi et al. (2020); Mattamala et al. (2024); Freißmuth et al. (2024); Chirici et al. (2023), while wheeled platforms enabled efficient ground-level mapping through sensor fusion and navigation algorithms Rossmann et al. (2010); Ferreira et al. (2023).

Jumping robots have also been explored for traversing cluttered terrain through rapid ballistic motion Hsiao et al. (2025); Kim et al. (2024). While effective at overcoming gaps and obstacles, these systems remain fundamentally ground-oriented, with trajectories that originate and terminate near the forest floor, limiting their ability to access or operate within the canopy.

More specialized systems adopted bioinspired climbing strategies. Gecko-inspired robots employed adhesive pads and tail-assisted reorientation to ascend vertical surfaces Siddall et al. (2021); Fukushima et al. (2021), while plant-inspired climbing robots used growth-based or soft adhesion mechanisms to climb trunks and artificial supports Hawkes et al. (2017); Sadeghi et al. (2017); Del Dottore et al. (2018); Fiorello et al. (2023); Del Dottore et al. (2024).

Despite these advances, such systems remained largely confined to trunks or near-ground structures. Their limited reach, slow ascent, and sensitivity to complex branch geometries prevented systematic access to the upper canopy. In evolutionary terms, these robots resemble early arboreal organisms capable of climbing but not yet adapted to navigate interconnected branch networks Cartmill (1985). We identify these systems as *sessile harvesters*. Much like orchids or ferns that grow on host trees, these robots are securely anchored to the trunk or a massive bough of a single mother tree.

#### 2.1.3 Aerial robots: flying visitors to the canopy

Aerial robots marked a significant evolutionary innovation, analogous to the emergence of flying and gliding animals. Multirotor drones equipped with cameras, LiDAR and multispectral sensors enabled high-resolution three-dimensional mapping, accurate localization and rapid spatial coverage of forest canopies Adão et al. (2017); Manfreda et al. (2018); Jaakkola et al. (2017); Pritchard et al. (2025) (Figure 1). Furthermore, canopy level approaches capturing trunks, leaves, tree crown and standing trees are considered for emergent tree diseases such as ash dieback Ho et al. (2022); Bates et al. (2025). Autonomous navigation and obstacle avoidance in cluttered environments were also achieved through bioinspired control strategies Cui et al. (2014); Chisholm et al. (2013); Zhou et al. (2022). For example, a palm-sized drone achieved collision avoidance through optical flow in real forested environment with complex shrubs Xiao et al. (2021).

**FIGURE 1:**
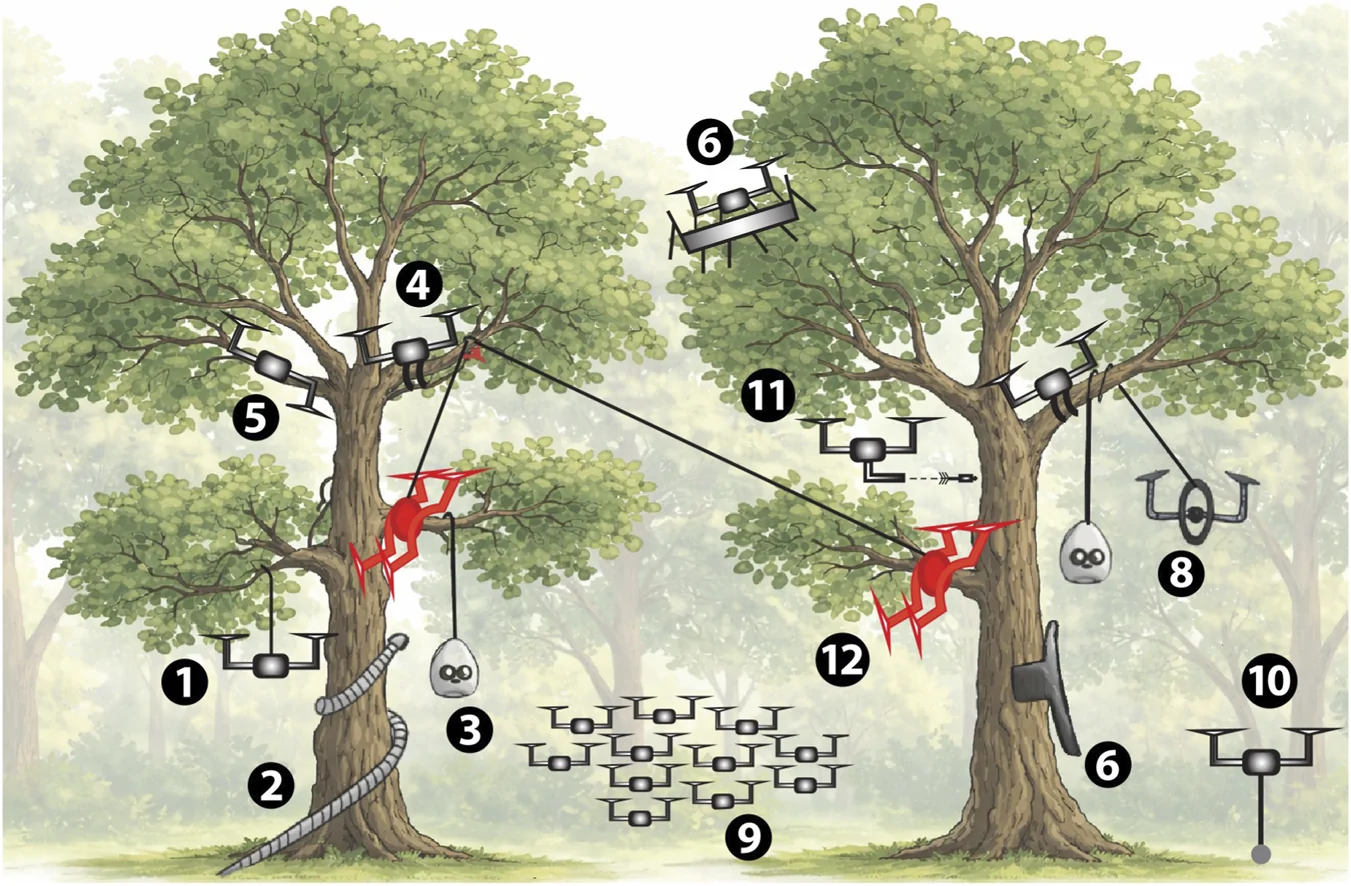
Examples of aerial and tree-climbing robotic systems: (1) Drone perching from above Nguyen et al. (2019a); Li et al. (2025b); (2) Growing tree-climbing robot Del Dottore et al. (2024); (3) AVOCADO: tether-perched bi-motor robot Kirchgeorg et al. (2024); (4) Drone perching from below Nguyen et al. (2023); Zheng and Hamaza (2024); (5) SLOTH: drone using its structure to perch on branches Zheng et al. (2023); (6) HEDGEHOG: drone with structure to “sit” on upper branches Kirchgeorg and Mintchev (2021) and vertical tree trunks Li et al. (2025a); (7) Fixed-wing drone crashing and perching on a trunk Askari et al. (2024); (8) Drone-ring tether perching a robot Lan et al. (2024); (9) Swarm of drones mapping the canopy Zhou et al. (2022); (10) Hopping drone Kim et al. (2024); (11) Sensor placement through dart shooting Farinha et al. (2020) (12) Romanello et al. (2026).

These systems reduced operational costs relative to manned aircraft while enabling frequent, repeatable surveys Manfreda et al. (2018); Besson et al. (2022). Beyond remote sensing, more recent work has begun to explore direct interaction with the canopy, including tasks such as sample collection and autonomous sensor placement Kocer et al. (2021). However, their reliance on continuous thrust imposed severe energetic constraints. Battery limitations restricted mission duration, particularly when carrying sensing payloads Floreano and Wood (2015); DJI (2023). As a result, aerial robots function primarily as transient visitors, briefly entering the canopy before retreating to recharge.

### 2.2 Perching as an intermediate evolutionary adaptation

Inspired by biological systems, perching mechanisms exploit mechanical intelligence to simplify control and interaction with complex environments Kovac (2016). To mitigate limited flight endurance, researchers have developed a wide range of bioinspired perching strategies, drawing from birds, bats, and arboreal insects to enable intermittent physical interaction and extended operational duration.

Microspine-based graspers enabled attachment to cylindrical branches Kirchgeorg and Mintchev (2021); Nguyen et al. (2019b), while avian-inspired claws and tendon-driven graspers replicated passive locking strategies Erbil et al. (2013); Thomas et al. (2014); Stewart et al. (2021); Roderick et al. (2021). Soft and origami-inspired mechanisms expanded adaptability to unknown geometries Zhang et al. (2019); Kim et al. (2018); Rubiales et al. (2021); Nguyen et al. (2023). At smaller scales, electrostatic adhesion has enabled perching on challenging surfaces such as overhangs Graule et al. (2016). Flapping-wing platforms further integrated bistable claws and Fin Ray-inspired grippers for rapid perching Broers and Armanini (2022), Broers and Armanini (2025); Zufferey et al. (2022); Hammad et al. (2024).

Parallel efforts introduced compliant bodies and collision-resilient structures to tolerate inevitable impacts within dense vegetation Mintchev et al. (2017); Dilaveroğlu and Özcan (2020); Patnaik et al. (2021). Soft wings, deformable rotors, and foldable frames improved survivability and interaction safety Mintchev and Floreano (2016); Floreano and Wood (2015); Nguyen et al. (2020); Sareh et al. (2018).

Despite their diversity, these systems share a common limitation: perching remains an event rather than a mode of existence. Perching mechanisms are often added as auxiliary payloads, increasing mass and control complexity, and typically support only localized, static interaction. Navigation between branches still relies on repeated takeoffs and landings, resulting in high energy expenditure and limited spatial persistence. Tethered systems address this precisely when intra-canopy movement matters most: by offloading weight support to the branch structure rather than continuous thrust, energy consumption drops by up to 2.5 × relative to free hovering Romanello et al. (2026), with further gains on longer paths where gravity would otherwise dominate the energy budget.

### 2.3 Tether robotics as a canopy-first adaptation

Tethered robots represent a departure from flight-dominant design and align more closely with a canopy-first evolutionary trajectory. By prioritizing anchoring, suspension, and tension-based interaction, tethers transform the canopy from an obstacle-filled volume into a navigable substrate.

Unlike perching or grasping strategies that rely on specific surface properties or precise branch geometry, tether-based interaction is largely independent of branch texture or shape. As long as sufficient space exists for deployment, tension in the tether can provide stabilization and support, reducing sensing and control requirements for reliable interaction with complex canopy structures. This inverts the tradeoff faced by free-flying systems: where flight handles open gaps easily but struggles in dense clutter, tethers are well suited to structurally rich environments but limited by gaps exceeding their reach Zeng et al. (2020), a constraint that weaver-deployed anchor networks are intended to address.

Ecologists have long used tether-based access methods such as the Single Rope Technique (SRT) to study canopy microclimates, epiphytes, and biodiversity Lowman et al. (2012). Unlike human SRT practitioners, robotic systems can sense tether tension and geometry in real time, enabling controlled trajectories and early detection of collision or failure that make pendular locomotion safe and repeatable. 1nspired by spiders and silk-producing organisms, tethered robots offer enhanced control, stability, and energy efficiency Hauf et al. (2023). Such systems have been explored across ground Kang et al. (2024), underwater Debruyn et al. (2020); Romanello et al. (2024), space Yu et al. (2018), glaciers Polzin et al. (2023) and forest domains Lan et al. (2024); Kirchgeorg et al. (2024); Wigner et al. (2025); Polzin et al. (2023).

SpiderMAV demonstrated tensile anchoring for low-power station keeping Zhang et al. (2017), while multimodal bicopter platforms and glacier monitoring systems illustrated the adaptability of tethered aerial robots Kirchgeorg et al. (2024). However, many existing systems still require manual deployment and remain limited in autonomously navigating dense canopies.

Two tethering modalities should be distinguished here: installation, the semi-permanent deployment of an anchor structure that, once placed, remains, much like a liana growing into the canopy; and reversible tethering, the repeated anchor-and-release cycle that constitutes a locomotion primitive. The discontinuous movement profile discussed in this work refers to the latter, reflecting the current mid-stage of canopy robot development. As tether infrastructure matures into pre-deployed networks spanning multiple trees, locomotion can shift from episodic discontinuous anchoring toward continuous exploitation of established lines, the progression captured by the weaver swarm and dynamic brachiator concepts introduced below.

While this work is motivated by forest environments, the principles of tether-based interaction is largely independent of branch surface texture, size, or geometry, since the tether requires only a stable point of contact for tension, unlike adhesion, friction-based gripping, or claw engagement, which all demand specific surface or geometric compatibility. Environments with sparse structural supports, constrained access, or requirements for persistent positioning, including industrial infrastructure, urban settings, and extraterrestrial terrains, present similar conditions in which continuous thrust becomes inefficient or impractical. These challenges are already being addressed in applications such as aerial inspection and repair of large-scale structures, including wind turbines Kocer et al. (2022), and autonomous sensor deployment in complex environments Stephens et al. (2022b), Stephens et al. (2022a); Farinha et al. (2020), as well as in emerging approaches to aerial additive manufacturing, where robots physically interact with and construct structures *in situ* Zhang et al. (2022); Kaya et al. (2025); Dams et al. (2025), Dams et al. (2024b), Dams et al. (2024a).

More broadly, these developments align with a growing shift toward robotic systems that actively participate in the formation and modification of their environment, as explored in early work on swarm-based robotic construction Stuart-Smith (2016).

From this perspective, tethered robots are better understood as structure-innate systems, whose operation emerges from anchoring, tension, and interaction with their surroundings rather than free-space mobility. The forest canopy thus serves not as a limiting application domain, but as a representative testbed for a broader class of structurally mediated robotic systems. Within this context, Romanello et al. (2026), a tether-perched aerial robot with compliant arms for multi-angle branch attachment, introduced in detail in Section 2.4, can be interpreted as an early instantiation of such structure-innate morphology.

As the resources of a single tree are exhausted, evolutionary pressure may demand a wider foraging radius. We identify this potential direction as robots develop into *pendular radial explorers*. This design trajectory is summarized in Figure 2, which organizes forest robotic systems according to their dominant mode of interaction with the environment, from ground-based and aerial approaches to emerging canopy-native, tether-mediated strategies.

**FIGURE 2:**
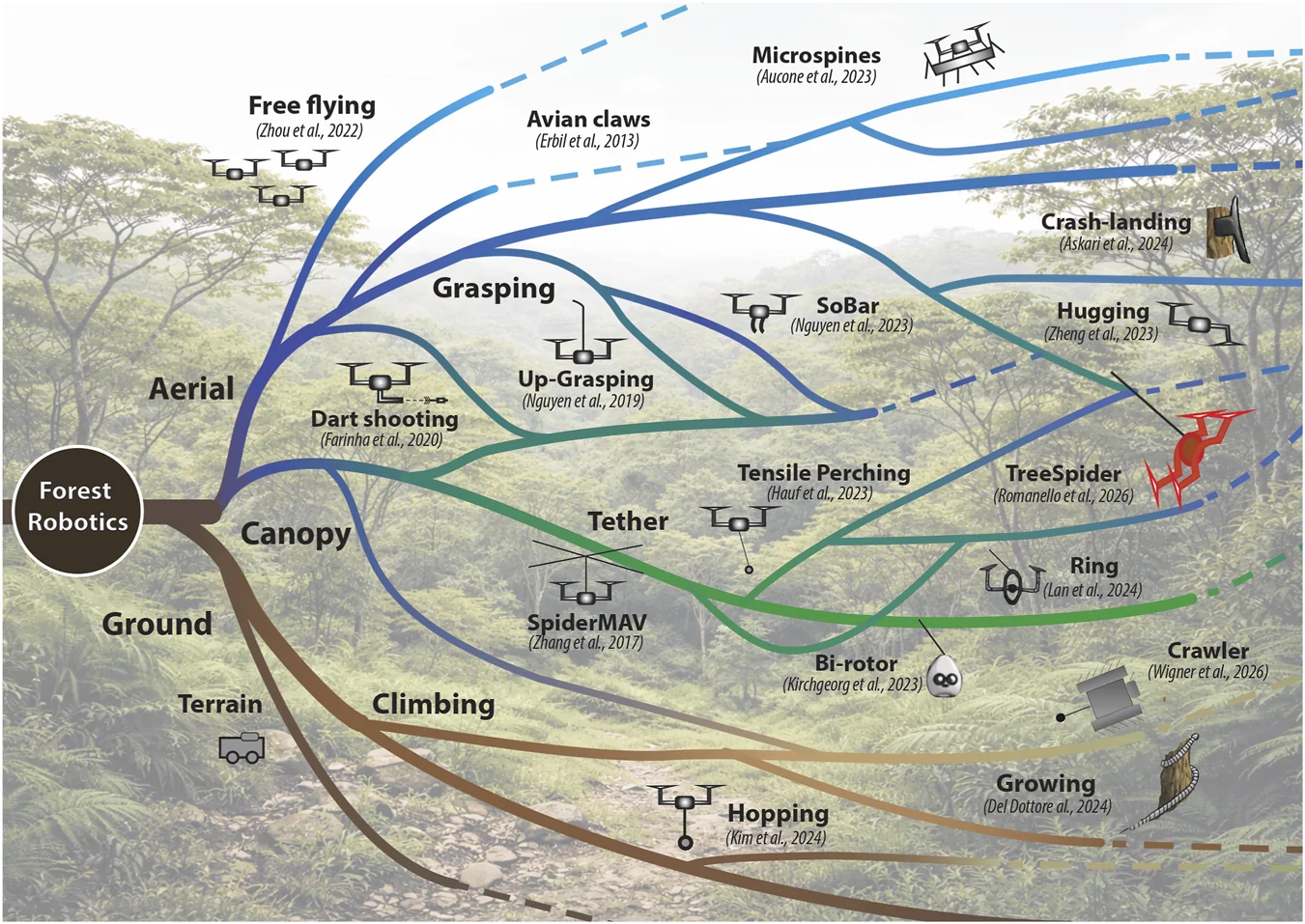
Design lineage of forest robotics as a tree diagram, not a strict phylogeny. Branch merging reflects engineering’s capacity to recombine innovations across distinct trajectories, unlike biological evolution. Branches ending abruptly indicate the current state of the art, not obsolescence. Early approaches relied on aerial and ground systems; more recent work introduces canopy-specialized, tether-mediated strategies.

### 2.4 TreeSpider as a canopy-innate robotic organism

Within this evolutionary framework, Romanello et al. (2026) can be interpreted not as a flight-centric platform, but as an early canopy-innate robotic organism. Building on prior tether-perching systems Lan et al. (2024), it integrates a ring-based morphology that decouples tether orientation from the vehicle frame, compliant perching arms for multi-angle branch interaction, and a dynamic tether that functions as a liana-like organ for anchoring, stabilization, and traversal.

Rather than relying on continuous thrust, TreeSpider adopts an intermittent locomotion strategy. Flight is used episodically to reposition anchors or transition between branches, while sustained operation is achieved through tether-mediated suspension and perching. This shifts the energetic burden from hovering to mechanical interaction with the environment, aligning with strategies observed in arboreal organisms that prioritize persistence over speed.

The tether plays a central functional role: it enables recovery from failed perching attempts, mitigates the consequences of collisions, and allows propellers to be slowed or stopped during anchored phases, reducing energy consumption, noise, and aerodynamic disturbance. As a result, physical contact becomes a recoverable and even advantageous mode of interaction rather than a failure condition.

TreeSpider therefore does not treat the canopy as a cluttered volume to be avoided, but as a structural network to be inhabited and anchored to. Branches serve simultaneously as supports, waypoints, and anchor points, enabling suspended locomotion that would be unattainable through flight or climbing alone. TreeSpider retains an aerial locomotor core: flight is the primary mechanism, and the tether functions as a safety and energy-saving aid during anchored phases, analogous to a rope in lead climbing. What distinguishes it from flight-dominant systems is not the absence of flight but a shift in how canopy interaction is sustained: extended anchored suspension punctuated by brief repositioning flights, rather than continuous hovering with occasional perching. TreeSpider is best understood as a transitional design, retaining aerial lineage while beginning to shift toward structure-mediated operation.

Extending this trajectory, the tether itself may evolve from a passive link into a distributed infrastructure. We describe this direction as the emergence of *weaver swarms*, in which multiple agents cooperatively deploy semi-permanent tensile networks. Within such environments, more advanced agents termed *dynamic brachiators*, a name drawn by analogy from the pendular swinging of biological brachiation though powered here aerodynamically rather than by limb musculature Fleagle (2013), could exploit these structures for rapid, energy-efficient movement, relying on temporary anchoring rather than continuous flight. Together, these systems suggest a progression from individual operation toward canopy-scale robotic ecologies.

Biological canopy-dwellers have not converged on tethering, but this reflects the constraints of evolutionary ancestry rather than a shortcoming of tethering itself: the required precursor structures may simply never have arisen, and intermediate transitional mechanisms may not have been viable under natural selection. Engineering faces none of these constraints. Robots can adopt tethering as a primary locomotor strategy precisely because they carry no ancestral baggage, which is the central advantage this manuscript argues for. The inverted tradeoff described earlier, where tethers excel in cluttered environments with gaps too large for direct grasping but too dense for easy flight, illustrates a genuine ecological niche that tethered robots can occupy as a terminal strategy rather than a transitional one.

### 2.5 Future evolution of canopy-innate robots

Interpreted through an evolutionary perspective, TreeSpider represents an early canopy-innate morphology rather than a final design. Just as biological organisms evolve increasing specialization under persistent ecological pressures, the robotics community has the possibility to design the next-generation of canopy robots, and more broadly structure-innate robotic systems, to diverge further from flight-dominant paradigms and toward deeper integration with arboreal structures.

#### 2.5.1 From passive to active anchoring

Current tether anchoring strategies, such as hooks and grapples, largely rely on passive attachment to external structures. A natural evolutionary step is the introduction of active anchoring robots capable of adapting to variable bark textures, branch compliance, and dynamic loads. These anchors, potentially realized as small drones or climbing robots, could employ actuated grasping, adaptive friction, or bioinspired fastening to regulate holding force in response to tension and motion. This would enable reliable multi point anchoring, support suspended manipulation, and reduce dependence on precise approach trajectories, effectively transforming the tether into a controllable robotic appendage rather than a passive constraint.

#### 2.5.2 Autonomy and long-term canopy residency

Sustained canopy presence introduces autonomy challenges that differ fundamentally from short-duration flight missions. Light availability is often limited beneath dense foliage, reducing the effectiveness of solar harvesting, while wind speeds are similarly attenuated under canopy cover, limiting wind-based harvesting independently, motivating exploration of alternative energy strategies, such as intermittent resurfacing, or distributed power-aware behaviors that exploit periods of low activity.

Autonomy must also extend beyond navigation to include long-term task scheduling, adaptive sensing strategies, and failure recovery without ground intervention. Rather than maximizing coverage speed, canopy-innate robots may prioritize persistence, opportunistic sampling, and slow, adaptive exploration over extended timescales.

#### 2.5.3 Sustainability and material ecology

A canopy-first robotic paradigm invites reconsideration of material choices and lifecycle impact. Robots designed to inhabit sensitive ecosystems for long durations should minimize environmental footprint not only during operation but also in failure scenarios. This motivates the use of biodegradable or bio-derived materials, modular architectures that allow partial recovery, and designs that degrade safely if retrieval is impossible Sethi et al. (2022); Wiesemüller et al. (2022); Romanello et al. (2025).

From an evolutionary standpoint, such constraints favor robustness through simplicity rather than redundancy through mass, as exemplified by sloths, whose passive grip strength and low-energy physiology enable long-term arboreal survival without complex active mechanisms Young et al. (2023).

#### 2.5.4 Ecological integration and animal–robot interaction

As canopy robots evolve toward long-term presence in forest ecosystems, their interaction with wildlife becomes a central design consideration. Early systems may operate primarily as passive observers, minimizing disturbance while monitoring biodiversity and ecological dynamics. However, deeper integration with canopy ecosystems may eventually enable new forms of animal–robot interaction, ranging from studying behavioral patterns to experimentally validating biological hypotheses about communication, movement, or collective dynamics Schmickl and Romano (2024).

Beyond observation, robots may also influence ecological processes, for example, by assisting in monitoring and managing invasive species, supporting restoration activities, or guiding animal behavior in controlled ways. Research on animal–robot systems has already demonstrated the potential for robots to interact with and influence animal groups, raising both opportunities and ethical challenges Barmak et al. (2023); Romano and Stefanini (2024).

Ultimately, the long-term vision may involve forms of ecological coexistence in which robots become stable components of canopy environments, interacting with animal communities without disrupting their natural dynamics. Achieving such integration requires careful consideration of ethics, ecological compatibility, and behavioral responses, ensuring that robotic systems support ecosystem understanding and conservation rather than unintentionally altering the environments they seek to study.

#### 2.5.5 Co-design with local ecological knowledge

Finally, future canopy robots should not be designed in isolation from the human communities that inhabit and steward forest ecosystems. Indigenous and local knowledge offers deep insights into canopy structure, seasonal dynamics, and minimal-impact interaction strategies that have evolved through long-term relationships between communities and forest ecosystems Reyes-García (2023).

Incorporating this knowledge into robotic design and deployment strategies can lead to systems that are not only technically effective but also culturally and ecologically compatible. Such co-design approaches represent a form of socio-ecological evolution, where robotic systems adapt not only to physical constraints but also to human-environment relationships.

#### 2.5.6 Beyond forests: structure-innate robotics across domains

While developed in the context of forest canopies, tether-based systems extend naturally to other structure-rich environments such as infrastructure inspection, construction, and extraterrestrial exploration, as highlighted by recent multi-environment testbeds for sustainability robotics Kaya et al. (2026). By offloading weight support to external anchors, tethers can reduce energy consumption by up to 2.5 × Romanello et al. (2026), enable drones to temporarily shut down propulsion while remaining suspended, and increase effective payload capacity by relieving the need to continuously counteract gravity.

From this perspective, tether robotics represents a specific instance of a broader class of structure-innate systems, in which performance emerges not from continuous flight but from anchoring, tension, and environmental integration.

#### 2.5.7 Toward canopy-wide robotic ecologies

Ultimately, we envision canopy robots evolving from single-agent systems into distributed, cooperative ecologies capable of sharing anchors, relaying energy or data, and collectively inhabiting forest canopies. In such a future, the canopy is no longer a region to be briefly visited, but a persistent operational domain, one in which robots behave less like flying machines and more like indigenous organisms of the treetops.

Tether infrastructure could also serve terrestrial robots, already optimized for surface navigation, rather than aerial agents alone. Weaver swarms would then perform a terraforming function, deploying tensile networks that turn a discontinuous canopy volume into substrate traversable by ground-optimized platforms Braithwaite et al. (2018). This trajectory raises serious ecological and ethical concerns, however: dense robotic infrastructure would alter the canopy for its existing inhabitants, from epiphytes to canopy-obligate fauna, and should be pursued only in dialogue with ecologists and the communities that steward these ecosystems.

## 3 Discussion

This Perspective argues that forest robotics has followed a predominantly ground-first and flight-dominant trajectory, in which the canopy is treated as an obstacle or sensing target rather than a structural environment. Across existing approaches, interaction with the canopy remains transient, relying on continuous thrust or discrete attachment events. Tether-based systems introduce a different paradigm: by enabling anchoring and tension-mediated interaction, they redefine locomotion as a sequence of attachment, loading, and release. In this view, performance emerges not only from actuation, but from the physical coupling between robot and environment, positioning tethered platforms as early examples of structure-innate robotic systems.

TreeSpider illustrates this transition by combining aerial mobility with sustained anchoring, but also highlights open challenges in autonomy, reliability, and scalability. Moving toward long-term canopy residency will require advances in active anchoring, energy-aware operation, and ecological integration. More broadly, the principles of structure-mediated interaction extend beyond forests to other environments where continuous flight is inefficient or impractical. This shift suggests a redefinition of robotic mobility, from movement through free space to operation through attachment and interaction with structure.

The shift from discontinuous to continuous locomotion is scale-dependent, reflecting the ratio of robot body and reach size to environmental feature size such as branch diameter and inter-branch gap. TreeSpider currently operates in a fully discontinuous regime, where each anchor is a discrete event. A near-term step toward continuity is roboticizing the anchor point itself, replacing the passive hook with an active crawling grasper capable of traversing branches continuously, as demonstrated at smaller scale in our prior work Wigner et al. (2025); a drone-mountable variant is a natural extension.

More broadly, the principles of structure-mediated interaction extend beyond forests to other environments where continuous flight is inefficient or impractical. This shift suggests a redefinition of robotic mobility, from movement through free space to operation through attachment and interaction with structure.

## Data Availability

The original contributions presented in the study are included in the article/supplementary material, further inquiries can be directed to the corresponding author.
